# Rasfonin promotes autophagy and apoptosis via upregulation of reactive oxygen species (ROS)/JNK pathway

**DOI:** 10.1080/21501203.2016.1170073

**Published:** 2016-04-06

**Authors:** Weijun Wang, Hui Sun, Yongsheng Che, Xuejun Jiang

**Affiliations:** a State Key Laboratory of Mycology, Institute of Microbiology, Chinese Academy of Sciences, Beijing, China; b University of Chinese Academy of Sciences, Beijing, China; c Beijing Institute of Pharmacology & Toxicology, Beijing, China

**Keywords:** Rasfonin, autophagy, apoptosis, ROS, JNK

## Abstract

Rasfonin is a fungal secondary metabolite demonstrating with antitumour effects. Reactive oxygen species (ROS) are formed as a natural by-product of the normal metabolism of oxygen and have important roles in cell signalling and homeostasis. Studies reported that many fungal secondary metabolites activated either autophagy or apoptosis through ROS generation. In former study, we revealed that rasfonin induced both autophagy and apoptosis, however, whether it promoted aforementioned processes via upregulation of ROS generation remains explored. In the current work, we demonstrated that rasfonin induced autophagy and apoptosis concomitant with a dramatically ROS production. N-Acetylcysteine (NAC), an often used ROS inhibitor, decreased both autophagic flux and caspase-dependent apoptosis by rasfonin. Flow cytometry analysis revealed NAC was able to reduce rasfonin-dependent apoptosis and necrosis. In methanethiosulfonate (MTS) assay, we observed that NAC significantly blocked rasfonin-induced cell viability loss. In addition, we found that rasfonin increased the phosphorylation of c-Jun NH2-terminal kinase (JNK), which was inhibited by NAC. SP600125, an inhibitor of JNK, reduced rasfonin-dependent autophagic flux and apoptosis. Moreover, we demonstrated that rasfonin inhibited the phosphorylation of both 4E-binding protein 1 (4E-BP1) and S6 kinase 1 (S6K1), two main substrates of mammalian target of rapamycin (mTOR). Collectively, rasfonin activated autophagy and apoptosis through upregulation of ROS/JNK signalling.

## Introduction

Macroautophagy (hereafter called autophagy) is a degradative process that involves delivery of cytoplasmic components, such as proteins, organelles and invaded microbes to the lysosome for digestion (Hippert et al. 2006). Autophagy has been found to be implicated in various human diseases and can either promote cell survival or cell death (Kroemer and Levine 2008; Gump et al. 2014). In different cellular contexts, a complex of signalling pathways controls the activation of autophagy (Zhu et al. 2007; Maiuri et al. 2010). Reactive oxygen species (ROS) are highly reactive oxygen free radical or non-radical molecules that are produced by multiple mechanisms in cells (Apel and Hirt 2004). These ROS are important signalling molecules that mediating many signal transduction pathways, playing critical roles in cell survival and death and participating in many diseases (Ray et al. 2012). Recently, ROS were demonstrated to promote starvation-induced autophagy, antibacterial autophagy and autophagic cell death (Scherz-Shouval and Elazar 2007). There is now an accumulating consensus that ROS controls autophagy in multiple contexts and cell types (Scherz-Shouval and Elazar 2007, 2011). Moreover, changes in ROS and autophagy regulation contribute to cancer initiation and progression (Tang et al. 2010). In tumour treatment, therapeutic drugs that increase ROS and autophagy were implicated in their mechanism for cell death (Ray et al. 2012).

For a long time, apoptosis was believed the sole form of programmed cell death during development, homeostasis and disease, whereas necrosis was considered as an unregulated and uncontrollable procedure. Growing evidence reveals that necrosis can also occur in a regulated manner (Elmore 2007). Based on morphology, three major types of programmed cell death have been coined: apoptosis, autophagic cell death and programmed necrosis (Eisenberg-Lerner et al. 2009). Under oxidative stress, ROS including free radicals, such as superoxide, hydroxyl radical and hydrogen peroxide are generated at high levels leading to cellular damage and cell death (Gump et al. 2014). This kind of cell death often involves induction of apoptosis through caspase activation. In macrophages, one study reported that ROS contribute to caspase-independent cell death (Yee et al. 2014). Therefore, in addition to autophagy, ROS is actively involved in the regulation of apoptosis.

Accumulating evidence has indicted that there are several molecular connections among autophagy, apoptosis and programmed necrosis (Eisenberg-Lerner et al. 2009). For cells undergoing persistent autophagy, hallmarks of apoptosis, such as caspase activation, necrotic cell death, organelles swelling and plasma membrane rupture, are often observed (Chu and Shatkin 2008). Depending on the cellular setting, the same proteins can regulate both autophagic and apoptotic processes. For example, p53, a potent inducer of apoptosis, also promotes autophagy via its target gene, damage-regulated modulator of autophagy (DRAM). Beclin 1, required for formation of the autophagic vesicles, was also found to interact with both Bcl-2 and Bel-xL (Swerdlow and Distelhorst 2007). Until now, three different types of interplays between autophagy and apoptosis have been suggested: autophagy can act as a partner, and antagonist or an enabler of apoptosis (Longo et al. 2008).

Generally, the mammalian target of rapamycin (mTOR), a 289-kDa serine/threonine protein kinase also known as fructose bisphosphatase-12/rapamycin-associated protein (FRAP), is a negative regulator of autophagy (Chiang and Abraham 2005). As a member of the PI3K-related kinase family, mTOR is found in two distinct complexes, mTORC1 and mTORC2, and regulates many aspects of cellular functions (Wullschleger et al. 2006; Zhu et al. 2007). mTORC2 can activate Akt, while mTORC1 is primarily activated by PI3K/Akt (Reiling and Sabatini 2008). Once activated by Akt, mTORC1 elicits a negative feedback loop to inhibit the activity of Akt (Harwood et al. 2008). mTORC1 phosphorylates two main substrates, ribosomal protein S6 kinase 1 (S6K1) and eukaryotic initiation factor 4E-binding protein 1 (4E-BP1) (Weisman et al. 2007).

In response to a variety of different stimuli, mitogen-activated protein kinases (MAPK) transducer signals from the cell membrane to the nucleus and involve in various intracellular signalling pathways that control a wide spectrum of cellular processes, including growth, differentiation and stress responses (Edick et al. 2007). MAPKs include extracellular signal-regulated kinase (ERK), c-Jun NH2-terminal kinase (JNK) and p38 MAPK (Gregory et al. 2004). Different from ERK pathway, JNK and p38 MAPK are weakly activated by growth factors, but respond strongly to stress signals, including tumour necrosis factor, interleukin-1, ionizing and UV irradiation, hyperosmotic stress and chemotherapeutic drugs (Heinrichsdorff et al. 2008). Activation of these kinases is strongly associated with apoptotic cell death induced by stress stimuli (Chu and Shatkin 2008). Recent studies reported that JNK also played a critical role in the regulation of autophagy (Goussetis et al. 2010).

Many fungal secondary metabolites were demonstrated to increase levels of cellular oxidative stress (Wu et al. 2012). 11ʹ-deoxyverticillin A is a member of a class of fungal secondary metabolites known as epipolythiodioxopiperazines (ETPs) and its toxicity to animal cell by generation of ROS via redox cycling (Zhang et al. 2011). And X15-2, another small-sized compounds, promotes autophagy through generation ROS (Xue et al. 2015).

In present study, we explored whether rasfonin could produce ROS and, demonstrated ROS played a critical role in rasfonin-dependent autophagy and apoptosis. Moreover, we revealed that JNK signalling functioned downstream of ROS to mediate rasfonin-induced autophagy and caspase-dependent apoptosis.

## Result

### Autophagy is involved in rasfonin-induced cell death processes

**Figure 1.**
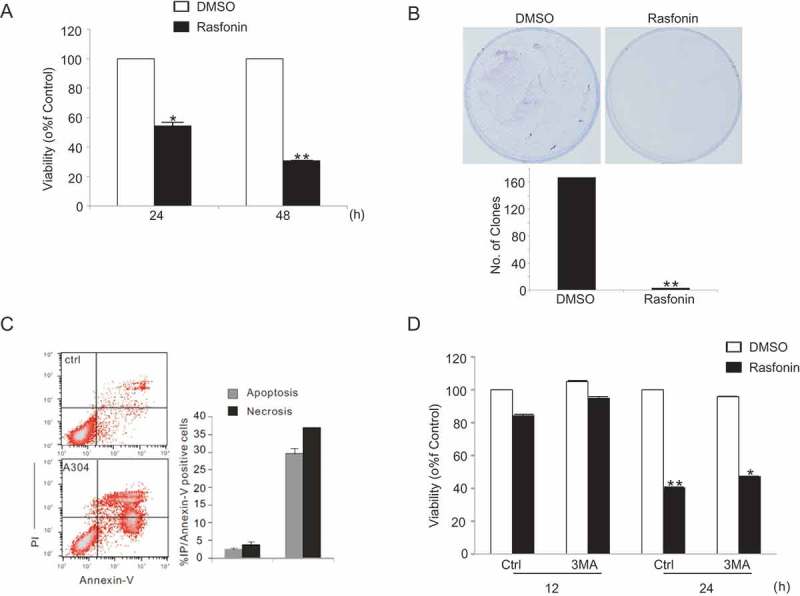
Autophagy is involved in rasfonin-induced cell death processes. (a) ACHN cells were treated with rasfonin with the concentration of 6 μM for 24 and 48 h. Cell viability was analysed by methanethiosulfonate (MTS) assay as described in Materials and Methods. The single asterisk denotes the group is statistically different from the control groups (*p* < 0.05), and double asterisk means *p* < 0.01. (b) Colony survival assays in ACHN cells were performed following the treatment of ACHN cells with rasfonin 1 μM for 14 d. Data represent the mean ± SD of three experiments, each performed in triplicate. (c) Following treatment of ACHN cells with rasfonin (6 μM) for 12 h, the apoptosis and necrosis induced were determined by flow cytometry. Apoptotic: AV positive and PI negative; necrotic: PI positive; AV: annexin V. The data are presented as mean ± SD from three independent experiments. (d) ACHN cells were treated with rasfonin with 6 μM for 12 and 48 h with the presence or absence of 3-MA (2 mM). Cell viability was analysed by MTS assay as described in Materials and Methods. The double asterisk denotes the group is statistically different from the control groups (*p* < 0.01).

Human renal cancer cell line ACHN was selected to detect rasfonin-induced cell death in the present study. As shown in Figure 1(a), rasfonin caused cell viability loss of ACHN cells in a time-dependent manner. In colony growth assay, rasfonin demonstrated to suppress cell growth remarkably (Figure 1(b)). Flow cytometry data revealed that the rasfonin-induced cell death of ACHN could be either apoptotic or necrotic (either necrosis or secondary necrosis) (Figure 1(c)). Interestingly, 3-methyladenine (3-MA), widely used inhibitor of autophagy (Kabeya et al. 2004), partially rescued rasfonin-induced cell viability loss (Figure 1(d)), suggesting that autophagy is involved in rasfonin-activated cell death processes.

### Rasfonin enhances autophagy and inhibits mTORC1 signalling

**Figure 2.**
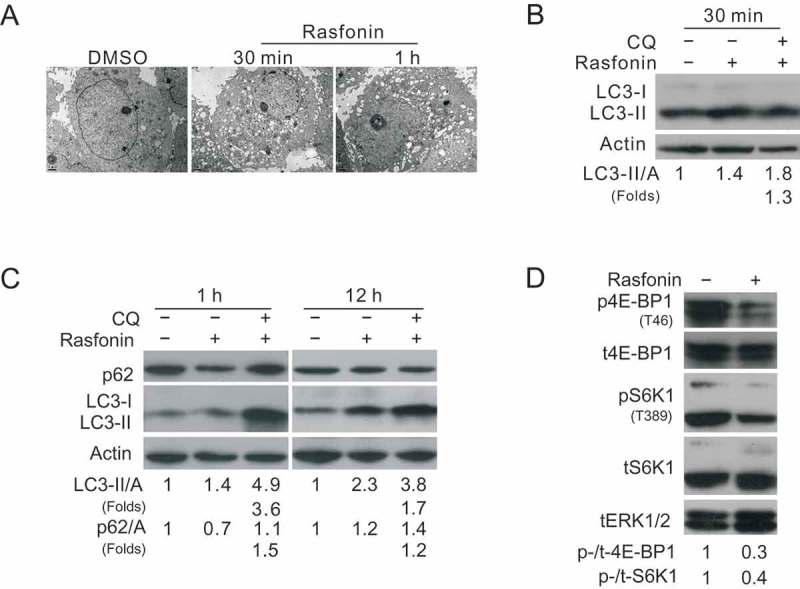
Rasfonin enhances autophagy and inhibits mTORC1 signalling. (a) Electron microscopy was used to detect the vacuoles in ACHN cells in the medium of rasfonin (6 μM) for 30 min and 1 h. (b) ACHN cells were treated with rasfonin (6 μM) for 30 min (c:1 h, 12 h) in the presence or absence of CQ (15 μM). The lysates of the cells were analysed by western blotting with the indicated antibodies. Actin was used as loading control. (d) ACHN cells were treated with rasfonin (6 μM) for 1 h and cell lysates were prepared and analysed by immunoblotting using the indicated antibodies, tERK1/2 was used as loading control.

Electron microscopy (EM), which is considered as one of the most convincing approaches to detect autophagy (Klionsky et al. 2012), is used to determine whether rasfonin induces autophagy or not. We found that rasfonin rapidly induced an obvious accumulation of membrane vacuoles in ACHN cells at the both 0.5- and 1-h time points (Figure 2(a)). In immunoblotting assay, rasfonin revealed to increase the ratio of LC3-II to actin, which is an indicator of autophagy (Kabeya et al. 2004), at 0.5-, 1- and 12-h time points. Chloroquine (CQ), which is known as inhibitor of autophagosome–lysosome fusion and often used in autophagic flux detection (Klionsky et al. 2012), further increased rasfonin-induced LC3-II accumulation, indicating that rasfonin can activate autophagic flux (Figure 2(b) and 2(c)). Moreover, we observed that rasfonin was able to promote the degradation of p62/SQSTM1 (Sequestosome 1), a selective substrate of autophagy and degraded when autophagy is activated (Figure 2(c)). Since the kinase activity of mTOR can be inferred by measuring the phosphorylation of its two substrates, S6K1 and 4E-BP1, we next examined the phosphorylation of S6K1 and 4E-BP1 in response to rasfonin stimulation. Expectedly, rasfonin demonstrated to decrease the phosphorylation of either S6K1 or 4E-BP1, suggesting that rasfonin triggered autophagic process by downregulation of mTORC1 signalling (Figure 2(d)).

### Rasfonin stimulates autophagy and apoptosis through rapidly ROS generation

**Figure 3.**
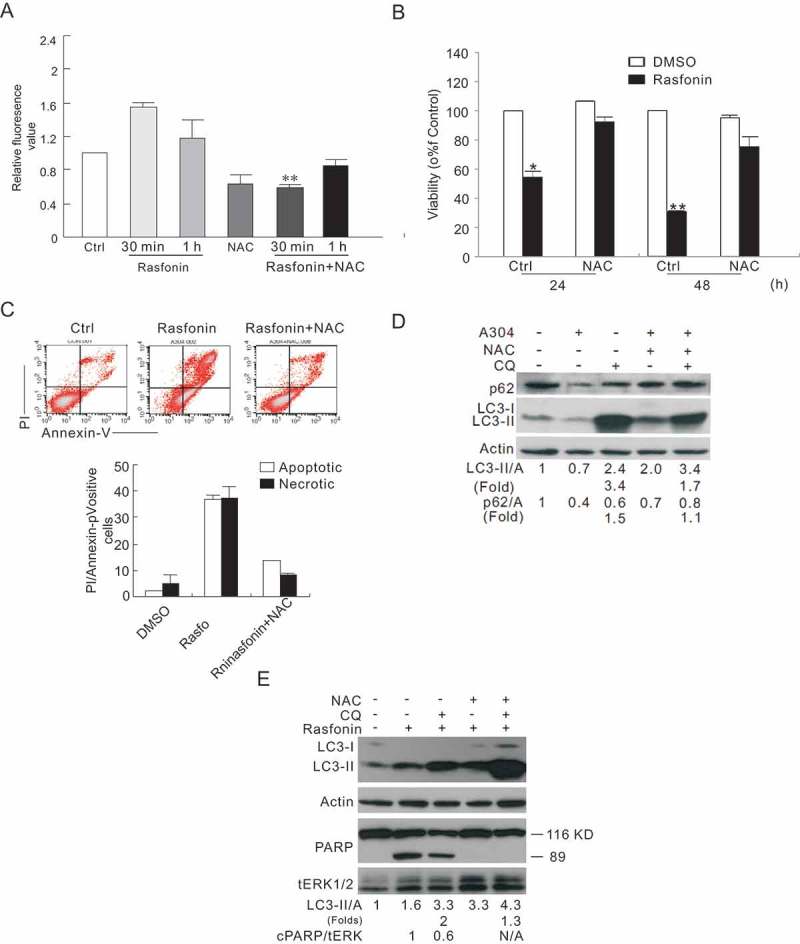
Rasfonin stimulates autophagy and apoptosis through rapidly ROS generation. (a) ACHN cells were treated with rasfonin (6 μM) in the presence or absence of NAC (50 μM) for 30 min and 1 h. Reactive Oxygen Species Assay Kit was used to detect the levels of reactive oxygen species (ROS) in ACHN cells with Multiscan Spectrum. (b) Following treatment of the cells with rasfonin (6 μM) for 24 h and 48 h with or without NAC (50 μM), and MTS assays was used to detect the cell viability above. (c) After treatment with rasfonin (6 μM) for 12 h, the apoptosis and necrosis induced were determined by flow cytometry. Apoptotic: AV positive and PI negative; necrotic: PI positive. For histogram results, the data are presented as mean ± SD from three independent experiments. (d) ACHN cells were treated with rasfonin (6 μM) in the presence or absence of NAC (50 μM) for 2 h, and cell lysates were prepared and analysed by immunoblotting using the indicated antibodies. Densitometry was performed for quantification and the ratios of LC3-II and p62 to actin are presented below the blots. The ratios were representative of at least three independent experiments. Densitometry was performed for quantification and the presence of cleaved PARP (cPARP) means apoptosis was induced. N/A: not available.

Overproduction of ROS caused damage to the cells and was involved in the regulation of either autophagy or apoptosis (Apel and Hirt 2004); thus, we next determine the participation of ROS in rasfonin-induced cell death processes. It demonstrated that rasfonin dramatically increased ROS production to a maximum extent at the 0.5-h time point (Figure 3(a)). N-Acetylcysteine (NAC), an often used ROS inhibitor, reduced rasfonin-induced ROS generation (Figure 3(a)). And rasfonin-induced cell death was suppressed in 24 h and 48 h (Figure 3(b)). Flow cytometry data revealed that NAC decreased rasfonin-dependent apoptosis and necrosis (Figure 3(c)), indicating that rasfonin activated above cell death pathways via mediating ROS production. Moreover, we observed that NAC attenuated rasfonin-induced autophagy as evaluating LC3-II accumulation and p62 degradation in the presence of CQ (Figure 3(d)). Although rasfonin decreased LC3-II levels at 2-h time point, yet, CQ was able to prevent LC3-II from degradation (Grumati et al. 2010; Klionsky et al. 2012), suggesting an enhanced autophagic flux (Figure 3(d)). Meanwhile, NAC also blocked the cleavage of PARP-1 (Figure 3(e)), a hallmark of apoptosis (Amé et al. 2004), and indicating that ROS is also involved in rasfonin-induced apoptotic process.

### Inhibition of JNK pathway attenuates both autophagy and caspase-dependent apoptosis by rasfonin

**Figure 4.**
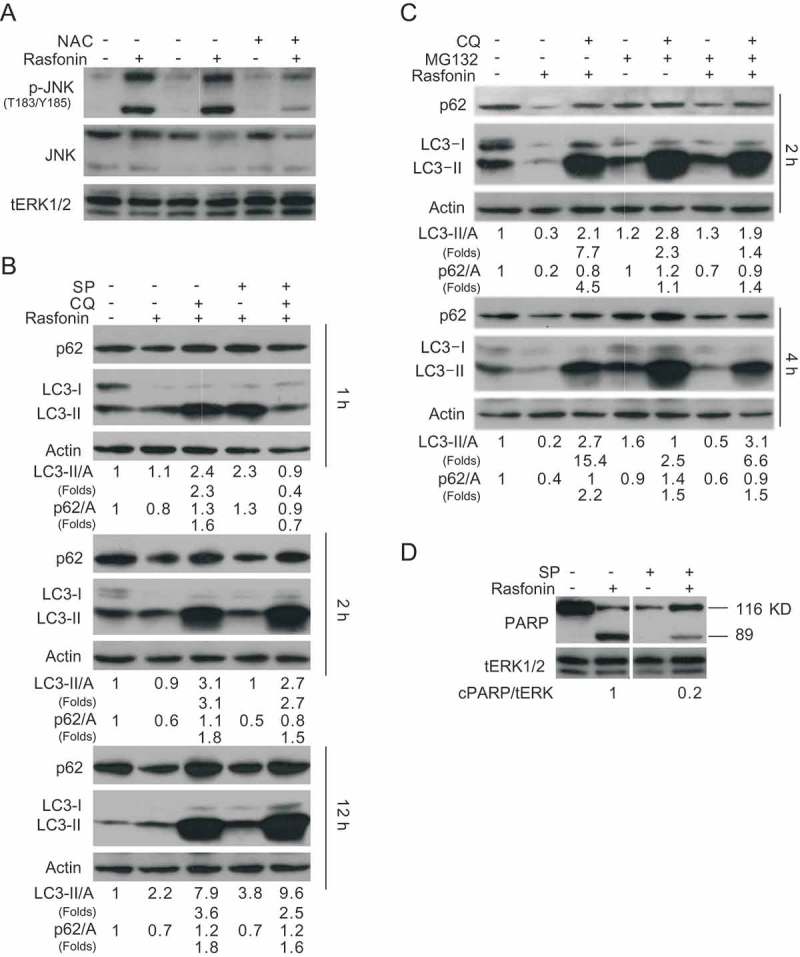
Inhibition of JNK pathway attenuated both autophagy and caspase-dependent apoptosis by rasfonin. (a) ACHN cells were treated with the combination of rasfonin (6 μM) and SP (30 μM) for 2 h and cell lysates were prepared and analysed by immunoblotting using the indicated antibodies, tERK1/2 was used as loading control. (b) Immunoblotting analysis was performed with the indicated antibodies following exposure to the combination of rasfonin (6 μM) and SP ((30 μM) in the presence or absence of CQ for 30 min, 1 h and 12 h. (c) ACHN cells were treated with the combination of rasfonin (6 μM) and MG132 (0.5 μM) for 2 h and 4 h and cell lysates were analysed by immunoblotting with the indicated antibodies. (d) Cell lysates were analysed by immunoblotting with the indicated antibodies following 12-h rasfonin (6 μM) in presence or absence of SP (30 μM) treatment. Densitometry was performed for quantification and relative ratios of cleaved PARP (cPARP) were shown below the blots.

JNK belongs to MAPK signalling pathways and is activated upon stimulation of ROS (Kim et al. 2010), and so does the downstream factor – NFκB. We observed that rasfonin increased the phosphorylation of JNK, which was inhibited by NAC (Figure 4(a)), confirming that ROS can act upstream of JNK. SP600125 (SP), an inhibitor of JNK, demonstrated to completely block rasfonin-dependent autophagy at the 1-h time point (Figure 4(b)). At both 2- and 12-h time points, it was able to decrease rasfonin-induced autophagic flux judging the LC3-II accumulation and p62 degradation in the presence of CQ (Figure 4(b)). MG132, a proteasome inhibitor and is often used to inhibit NFκB (Ko et al. 2010; Zanotto-Filho et al. 2012) attenuated rasfonin-induced autophagy as evaluating LC3-II accumulation and p62 degradation in the presence of CQ (Figure 4(c)). Moreover, we found that SP inhibited the cleavage of PARP-1 by rasfonin (Figure 4(d)). Aforementioned results indicated that JNK functioned downstream of ROS and played a critical role in the regulation of rafonin-induced either autophagy or caspase-dependent apoptosis.

## Discussion

Rasfonin, a fungal secondary metabolite, stimulates autophagy and apoptosis; however, it remains unknown whether rasfonin can promote above cell death processes through ROS. In this study, we clearly revealed that rasfonin induced rapidly generation of ROS, which was likely to mediate rasfonin-dependent autophagy and apoptosis via JNK signalling pathway.

Cancer cells produce higher levels of ROS than normal cells, and this leads to cancer progression (Hart et al. 2015). ROS are important signalling molecules that mediate many signal transduction pathways and benefit for cellular survival (Focaccetti et al. 2015); however, the overproduction of ROS damage cell by activation of apoptosis or necrosis. Growing evidence reveal that ROS also play an important role in the regulation of autophagy (Farah et al. 2016). Consistent with former study, here, we found that ROS are critical for rasfonin-dependent autophagy, necrosis and apoptosis. Huang et al. (2011) reported that ROS regulated autophagy through distinct mechanisms depending on cell types and stimulation conditions. In cancer treatment, while therapeutic drugs that augment ROS and autophagy have been implicated in their mechanism for cell death, other therapeutic drugs that generate ROS and promote autophagy seem to have a protective effect (Focaccetti et al. 2015; Koo et al. 2016). Concerning rasfonin, we found that, through ROS, it induced both autophagy and apoptosis. Immunoblotting data demonstrated that NAC abolished rasfonin-induced PARP-1 cleavage, whereas flow cytometry results indicated that NAC only partially decreased rasfonin-dependent apoptotic cell death. Therefore, we speculated that rasfonin possibly activated caspase-independent apoptosis. Collectively, it is reasonable to assume that, through induction of ROS, rasfonin could undergo cell death via multiple pathways.

Many signalling pathways have been found to regulate autophagic process (Jung et al. 2010), such as Adenosine 5′-monophosphate (AMP)-activated protein kinase, Akt/mTOR and MAPK, etc. MAPKs include ERK, JNK and p38 MAPK, and control a wide spectrum of cellular processes (Kim et al. 2008a). Accumulating evidence indicated that MAPKs actively participated in the regulation of autophagic process (Peter et al. 2010). In Parkinson’s and Lewy body diseases, human tissue study supports a role for ERK/MAPK in the regulation of autophagy (Jung et al. 2010). In colorectal cancer cells, a novel cell type-specific role of p38α MAPK is found to control and mediate autophagy (Kim et al. 2008a). Either autophagy or apoptosis has been found to be regulated by JNK-mediated Bcl-2 phosphorylation (Kim et al. 2008b). Wei et al. reported that JNK1-mediated Bcl-2 phosphorylation interferes with its binding to the proautophagy BH3 domain-containing protein Beclin 1, and had a dual role in autophagy and apoptosis regulation (Wei et al. 2008). Similar to their observation, we demonstrated that rasfonin was able to activate JNK signalling pathway, and ROS functioned upstream of JNK to regulate both apoptosis and autophagy by rasfonin.

In conclusion, rasfonin induces autophagy through oxidative stress/JNK signalling, which provides a novel mechanism for this fungal secondary metabolite-activated cell death processes. These enriched the machinery and broaden our understanding of fungal secondary metabolite-induced autophagy, apoptosis as well as necrosis.

## Materials and methods

### Chemicals and antibodies

Chloroquine diphosphate salt (CQ, C6628), N-acetyl-L-cysteine (NAC, A7250), SP600125 (S5567), MG132(M8699) and polyclonal antibodies against LC3 (L7543) were purchased form Sigma-Aldrich (St. Louis, MO, USA). Antibody of p62 (sc-28359) was acquired from Santa Cruz Biotechnology (Santa Cruz, CA, USA). Antibodies against PARP-1 (9542), p44/42 MAPK (total-Erk1/2, 9102), phospho-p70S6 kinase (Thr389, 9205), p70S6 kinase (S6K1, 9202), phospho-4E-BP1 (Thr37/46, 2855), phospho-SAPK/JNK (T183/Y185, 9521) and SAPK/JNK (9525) were purchased from Cell Signaling Technology (Beverly, MA, USA). And total 4E-BP1 (ab32130) was purchased from Abcam (Burlingame, CA, USA). Antibody against actin (TA-09) was obtained from Zhongshan Jinqiao Biocompany (Beijing, China). Methanethiosulfonate reagent powder (G1111) was acquired from Promega Corporation (Madison, WI, USA).

### Cell culture and western blot analysis

ACHN (human renal cancer cell line) were grown in Dulbecco modified Eagle medium (DMEM) medium containing 10% foetal bovine serum (GIBCO) and 1% antibiotics. Cells were grown to 70–80% and treated with varieties of compounds for indicated time. Whole cell lysates were prepared with lysis using Triton X-100/glycerol buffer, containing 50 mM Tris-HCl, pH 7.4, 4 mM ethylene diamine tetraacetic acid, 2 mM ethylene glycol tetraacetic acid and 1 mM dithiothreitol, supplemented with 1% Triton X-100, 1% sodium dodecyl sulfate (SDS) and protease inhibitors and then separated on a SDS-polyacrylamide gel electrophoresis gel (13, 10 or 8% according to the molecular weights for the proteins of interest) and transferred to polyvinylidene fluoride membrane. Immunoblotting was performed using appropriate primary antibodies and horseradish peroxidase-conjugated suitable secondary antibodies, followed by detection with enhanced chemiluminescence (Pierce Chemical).

### Cell viability assay (MTS)

Cells were plated in 96-well plates (5000–10,000 cells per well) in 100 µl complete culture medium. After overnight culture, the medium was replaced with complete medium that was either drug-free or contained rasfonin or other chemicals. The cells were cultured for various periods and cellular viability was determined with CellTiter 96 Aqueous Non-Radioactive Cell Proliferation Assay (Promega).

### Colony growth assay

Cells were seeded at a concentration 300 cells/ml and cultured for 2 weeks to allow colony growth in the presence or absence of the indicated concentration of rasfonin. Pictures were taken after 4% paraformaldehyde fixation and trypan blue stain, and then the numbers of colony were calculated by Image J.

### Flow cytometry assay

ACHN cells were treated with the indicated compounds, then trypsinised and harvested (keeping all floating cells), washed with phosphate buffer saline (PBS) buffer, followed by incubating with a fluorescein isothiocyanate-labelled annexin V (FITC) and propidium iodide (PI) according to the instructions of an Annexin-V-FITC Apoptosis Detection Kit (Biovision Inc., K101-100) and analysed by flow cytometry (FACSAria, Becton Dickinson). Percentages of the cells with annnexin V positive and PI negative stainings were considered as apoptotic, whereas PI-positive staining was considered to be necrotic.

### Electron microscopy

Electron microscopy was performed as described. Briefly, samples were washed three times with PBS, trypsinised, and collected by centrifuging. The cell pellets were fixed with 4% paraformaldehyde overnight at 4°C, postfixed with 1% OsO4 in cacodylate buffer for 1 h at room temperature (RT) and dehydrated stepwise with ethanol. The dehydrated pellets were rinsed with propylene oxide for 30 min at RT and then embedded in Spurr resin for sectioning. Images of thin sections were observed under a transmission electron microscope (JEM1230, Japan).

### Reactive Oxygen Species Assay Kit

DCFH-diacetate (DA) passively diffuses into cells and is deacetylated by esterases to form nonfluorescent 2′, 7′-dichlorofluorescein (DCFH). In the presence of ROS, DCFH reacts with ROS to form the fluorescent product DCF, which is trapped inside the cells. Cells were plated in 96-well plates (20,000–30,000 cells per well) in 100 µl complete culture medium. After overnight culture, the culture medium was first removed and the cells were washed three times with PBS, DCFH-DA, diluted to a final concentration of 10 μM with DMEM/F12, was added to cultures and incubated for 20 min at 37°C. The fluorescence was read at 485 nm for excitation and 530 nm for emission with a fluorescence plate reader (Genios, TECAN). The increase value compared to control was viewed as the increase of intracellular ROS.

### Statistical analysis

Normally distributed data are shown as mean ± SD and were analysed using one-way analysis of variance and the Student–Newman–Keuls post hoc test.
